# Effects of mTOR-Is on malignancy and survival following renal transplantation: A systematic review and meta-analysis of randomized trials with a minimum follow-up of 24 months

**DOI:** 10.1371/journal.pone.0194975

**Published:** 2018-04-16

**Authors:** Sebastian Wolf, Verena S. Hoffmann, Antje Habicht, Teresa Kauke, Julian Bucher, Markus Schoenberg, Jens Werner, Markus Guba, Joachim Andrassy

**Affiliations:** 1 Department of General, Visceral and Transplant Surgery, Ludwig-Maximilian’s University, Munich, Germany; 2 Department of Visceral and Transplant Surgery, Augsburg Hospital, Augsburg, Germany; 3 Institute of Medical Information Sciences, Biometry and Epidemiology (IBE), Ludwig-Maximilian’s-University, Munich, Germany; 4 Helmholtz Center Munich, German Research Center for Environmental Health, Munich, Germany; 5 Transplant Center, University Hospital Grosshadern, Ludwig-Maximilian’s University, Munich, Germany; Universidad de Navarra, SPAIN

## Abstract

**Background:**

mTOR-Is positively influence the occurrence and course of certain tumors after solid organ transplantation. The effect of mTOR-Is on the overall incidence of tumors irrespective of their origin is not entirely clear. Furthermore, conflicting data have been shown on mortality under mTOR-Is.

**Methods:**

The current literature was searched for prospective randomized controlled renal transplantation trials. There were 1415 trials screened of which 13 could be included (pts. = 5924). A minimum follow-up of 24 months was mandatory for inclusion. Incidence of malignancies and patient survival was assessed in meta-analyses.

**Results:**

The average follow-up of all trials was 40.6 months. Malignancy was significantly reduced under mTOR-Is compared to CNIs (RR 0.70, CI 0.49–0.99, p = 0.046). This effect remained stable when combined with CNIs (RR 0.58, CI 0.34–1.00, p = 0.05). When NMSCs were excluded the risk for malignancy remained significantly reduced under mTOR-I therapy (mono and combi) (RR 0.43, CI 0.24–0.77, p = 0.0046). Graft survival was minimally decreased under mTOR-Is (RR 0.99, CI 0.98–1.00, p = 0.054). This effect was abrogated when mTOR-Is were combined with CNIs (RR 0.99, CI 0.97–1.02, p = 0.50). Patient survival was not different (RR 1.00, CI 0.99–1.01, p = 0.54).

**Conclusions:**

Posttransplant patients have a lower incidence of malignancy when treated with an mTOR-I no matter if it is used in combination with CNIs or not. This beneficial effect remains significant even when NMSCs are excluded. With currently used mTOR-I-based regimen patient and graft survival is not different compared to CNI therapies.

## Introduction

The number of transplants and the alive transplant population is growing. In the US, an increase of 9.2% was encountered for annually performed transplants from 2010 to 2015 (OPTN data). This has implications for the medical system. According to an USRDS based analysis around 15.6% of renal transplant recipients are expected to die within the first three years after renal transplantation, of these 76.3% with a functioning graft representing 46.8% of all graft losses [1]. Most of these patients die of cardiovascular problems, followed by infections and malignancy. The significance of malignancy increases over time. Responsible for only 3.6% of the deaths with functioning graft after the first year post transplantation it increases to 16% within years 2–5 post transplantation approaching the level of infections [2].

Altogether, the incidence of post-transplant malignancies is increased 2- to 4-fold compared to the general population and tumors often show a more aggressive phenotype under immunosuppression [3–5]. It is a complex field with important different aspects: 1. Dependence on the tumor entities: Certain skin tumors are amongst those with the steepest increase under immunosuppression. Also, tumor incidence seems particularly high for infection-related tumors, i.e. lymphomas, cancers of anus, vulva, Kaposi etc. The vast majority of infection-unrelated tumors is also increased but to a lesser extent while some other tumors, i.e. breast, prostate etc. do not show an increased incidence [6–8]. 2. Correlation of the tumors with the transplanted organs: some tumors occur more often with certain organ transplants (liver cancer with liver transplantation) [9]. 3. Influence of the immunosuppressive drugs: it has been known for a long time that immunosuppressive therapy itself poses a risk for the development of certain tumors [10, 11]. Ensuing experimental work could confirm this finding especially for Azathioprine and CNIs [12, 13]. CsA is classified as carcinogenic by the International Agency for Research on Cancer [14].

Hence, big hopes were raised when anti-tumor activity could be attributed to mTOR-Inhibitors [15]. Since then many reports have appeared comparing Calcineurin- and mTOR-Inhibitors in every possible combination assessing not only the immunological potency but also looking closely at the tumor incidence.

Mirroring the complexity of this field the findings with respect to tumor incidence or recurrence have been inconsistent. Treatment of some tumor entities with mTOR-Is, i.e. Kaposi sarcoma, has been shown highly efficacious [16]. In patients with hepatocellular carcinoma the effect was much less convincing and especially in advanced stages of tumor disease overall and recurrence free survival remained unaffected by mTOR-Is [17, 18]. Nonetheless, intense research in the oncological field resulted in the approval of mTOR-Is for a variety of malignancies, i.e. renal cell carcinoma, subependymal giant cell astrocytoma, breast cancer, and progressive neuroendocrine tumors of pancreatic origin [16, 19].

In the meantime worrisome data have been published indicating a negative mTOR-I effect on patient survival [20–23].

Altogether it remains unclear to this date if mTOR-Is should be used to reduce the overall incidence of cancer after solid organ transplantation.

## Materials and methods

### Identification of the eligible trials

Full reports of controlled prospective trials were searched via PubMed (http://www.ncbi.nlm.nih.gov), ScienceDirect (http://www.sciencedirect.com) and the Cochrane Central Register of Controlled Trials (http://www.mrw.interscience.wiley.com/cochrane/cochrane_clcentral_articles_fs.html) up to April 2017 using the following terms: mTOR-inhibitor OR sirolimus OR everolimus AND transplant AND malignancy or cancer. The search was performed by two reviewers (S. W., J. A.).

### Inclusion criteria

Only prospective randomized multicenter and two single center renal transplantation trials were included starting 2002. The quality of the trials was assessed using the Jadad-score (minimum score of 2) and ITT-analysis. These trials were required to have had at least two treatment arms, one with an mTOR-I based immunosuppression either with or without a CNI and one arm containing an mTOR-I free CNI-based immunosuppression. The mTOR-I had to be introduced within 3 months after the transplantation as this approach is commonly used in randomized trials. This selection criterion ensured a long follow-up under mTOR-Is with only minimal CNI exposure. The retrieved trials were screened for information on posttransplant malignancies, graft and patient survival. When several publications showed the same cohort of patients, the information was summarized. Trials were only included when a minimum follow-up of 24 months existed.

Screening and inclusion of the articles was performed by two reviewers (S. W., J. A.).

### Data analysis

To summarize the available evidence, we calculated relative risks (RRs) for the incidence of posttransplant malignancies, graft and patient survival under CNI- and mTOR-I-based immunosuppression. If no malignancy was observed in a study arm 0.5 cases were added to both study arms to facilitate the calculation of the RR. If the incidence in both study arms was zero the incidence was set to 1% to receive a RR of 1. Publication bias was assessed by plotting study results against precision of the study (funnel plots) and the according regression tests [24]. Between study heterogeneity was examined using Q-test for heterogeneity and the I^2^ statistic [25]. Accounting for possible heterogeneity between the studies, we fitted random effects models to derive pooled estimators of the natural logarithms of the RR using the restricted maximum-likelihood estimator [26]. Standard errors were estimated using incidences and number of patients per group. All calculations were performed using the *metafor* package in the statistical software package R (version 2.14.2). P values below 0.05 were considered significant and all confidence limits were on the 95% level.

### Data extraction and methodologic quality

The following data were extracted from eligible articles by two reviewers (S.W., J.A.): induction therapy, number of patients per treatment arm, mTOR-I dose, start of mTOR-I treatment post transplantation, graft and patient survival, trough levels, follow-up period, description, type and incidence of events of posttransplant malignancy, and statistical analysis of the posttransplant malignancy under mTOR-Is, mTOR-Is in combination with CNIs and CNIs.

Methodological quality was assessed by three reviewers (S.W., J.A., V.H.) using the Jadad score and Intention to treat (ITT) analysis [27, 28]. The Jadad score addresses the items randomization, blinding and description of drop outs and withdrawals. The score ranges from 0 to 5. A score of at least 3 is being considered to be consistent with sound methodological quality. ITT was considered another important aspect for methodological quality assessment [29]. In addition, Cochrane Collaboration’s tool was used to further reduce the risk of bias [30].

## Results

### Included studies

**Fig 1:**
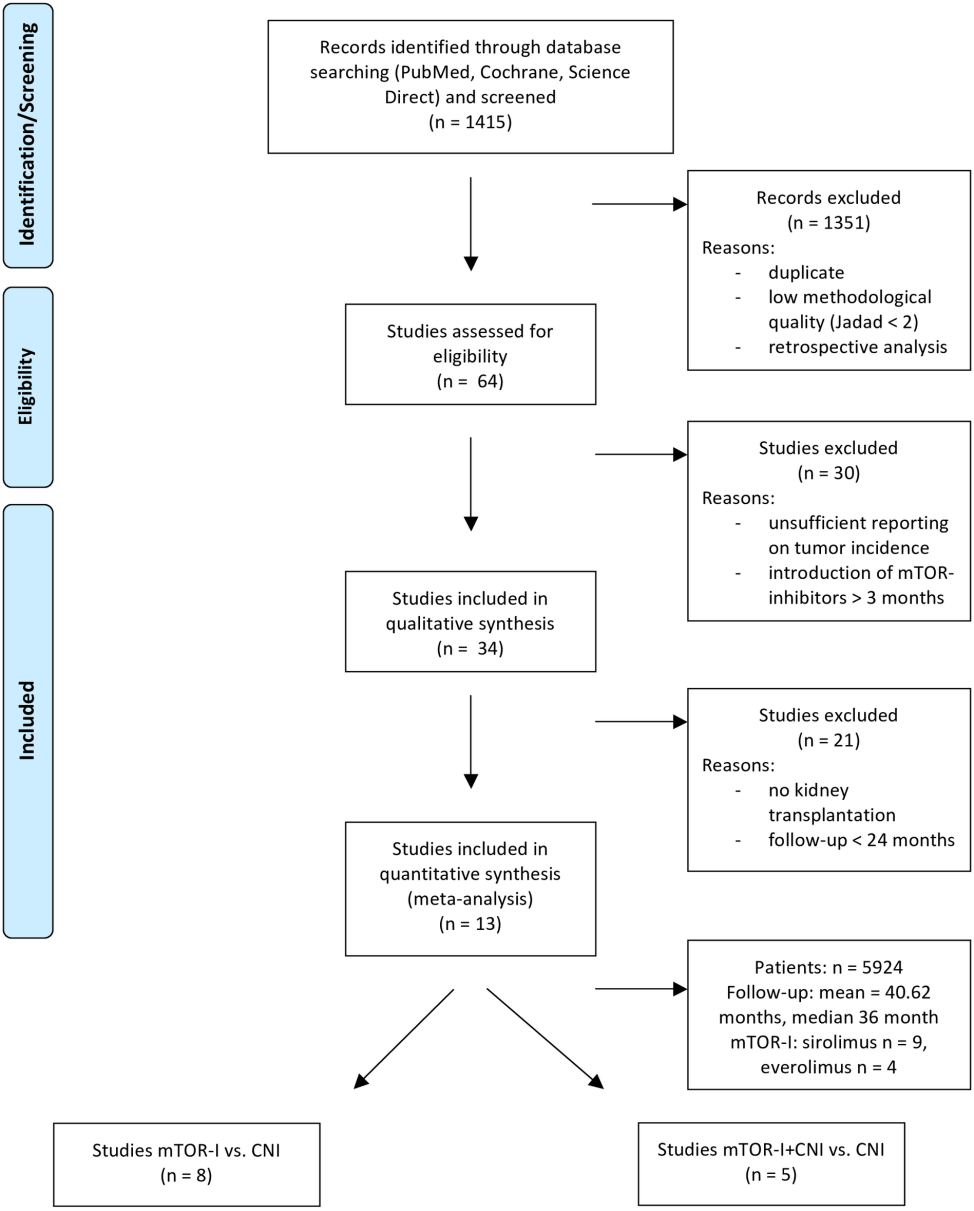
Flowchart of the selection of articles. *From*: Moher D, Liberati A, Tetzlaff J, Altman DG, The PRISMA Group (2009). *P*referred *R*eporting *I*temsfor *S*ystematic Reviews and *M*eta-*A*nalyses: The PRISMA Statement. PLoS Med 6(6): e1000097. doi:10.1371/journal.pmed1000097 **For more information, visit** www.prisma-statement.org.

The literature search produced 1415 studies, of which 13 met the inclusion criteria. Thus, a total number of n = 5924 patients were included (Fig 1). The average follow-up was 40.62 months. The trials were only on kidney transplantation. Eight trials compared mTOR-I with CNI treatment (S1 Table) and n = 5 mTOR-I+CNI vs. CNI (S2 Table). Of these thirteen trials, 9 RCTs used Sirolimus (SRL) and 4 Everolimus (EVRL). We only included studies with introduction of the mTOR-I within three months post transplantation. Mostly the mTOR-I was introduced de novo or very early (within the first month; n = 10). The majority used either monoclonal or polyclonal antibodies as induction therapy (n = 11).

Only longterm analyses on tumor incidence with and without NMSCs were performed. Furthermore, patient survival and graft survival censored for death were analyzed. The shortest follow-up duration was 24 months.

### Methodological quality

Eleven of the 13 RCTs were considered to be of good methodological quality according to the Cochrane Collaboration’s tool (S1 and S2 Figs) and the Jadad score (≥3) (S1 and S2 Tables). The remaining 2 had a Jadad-score of 2 due to unclear drop out reporting.

### Tumor incidence post transplantation—mTOR-I vs. CNI

**Fig 2:**
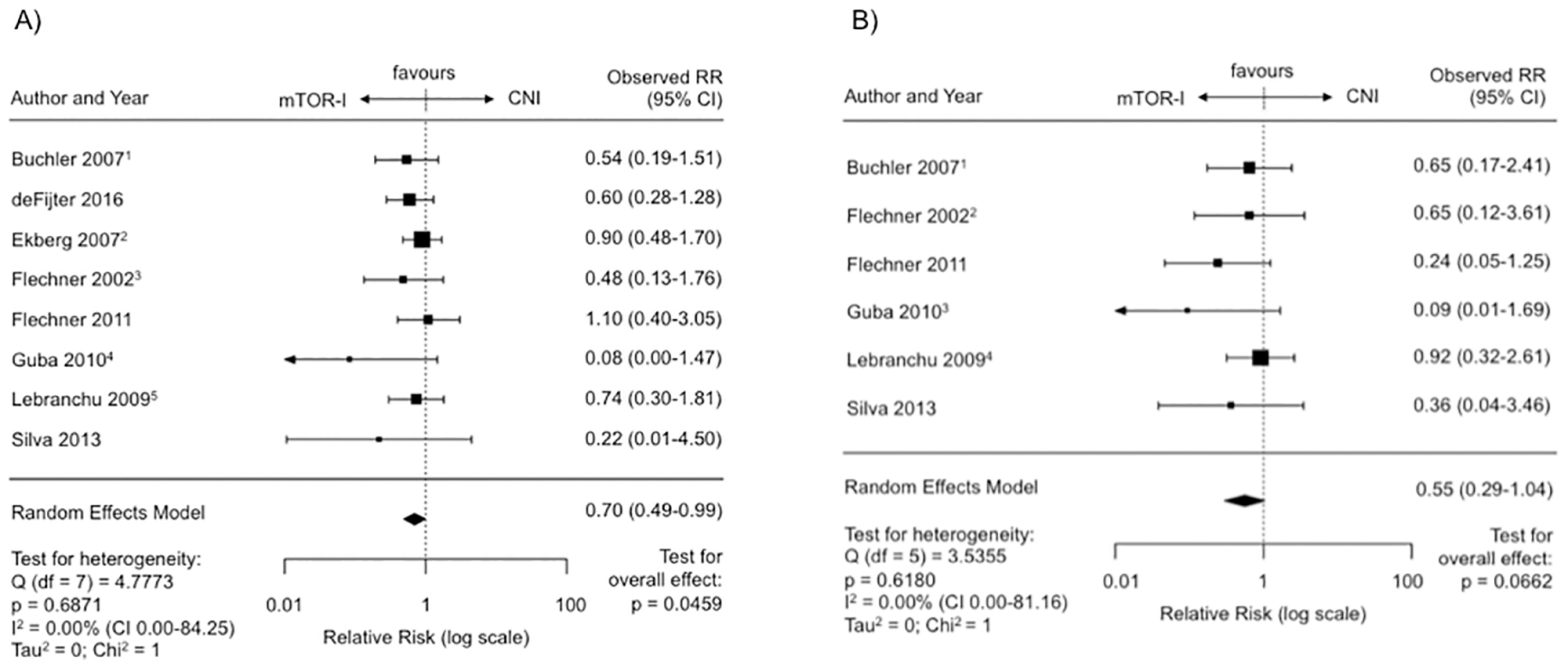
Malignancies on mTOR-I vs. CNI treatment post transplantation. (A) Forest plot indicating the relative risk of the occurence of malignancies. ^1^ Latest update by Gatault et al. in 2016. ^2^ Latest update by Ekberg et al. in 2009. ^3^ Latest update by Flechner et al. in 2007. ^4^ Latest update by Guba et al. in 2012. ^5^ Latest update by Lebranchu et al. in 2011. (B) Forest plot indicating the relative risk of the occurence of malignancies excluding NMSC’s. ^1^ Last update by Gatault et al. in 2016. ^2^ Last update by Felchner et al. in 2007. ^3^ Last update by Guba et al. in 2012. ^4^ Last update by Lebranchu et al. in 2011.

Treatment with an mTOR-I (n = 8, SIR = 7, ERL = 1) showed a significantly reduced risk for posttransplant malignancies compared to CNI treatment (RR 0.70, CI 0.49–0.99, p = 0.046) (Fig 2A). The regression test for funnel plot asymmetry was significant (p = 0.014) and the funnel plot itself reveals some asymmetry in favor of mTOR-I treatment upon visual inspection (S3 Fig). No heterogeneity between the studies was observed (I^2^ = 0.0%, Q-Test for heterogeneity: p = 0.69).

The analysis excluding NMSCs (n = 6) also revealed a reduced relative risk for the tumor incidence under mTOR-I treatment (RR 0.55, CI 0.29–1.04, p = 0.066) (Fig 2B). There was no heterogeneity observed between the studies (I^2^ = 0.0%, Q-Test for heterogeneity: p = 0.62).

### Tumor incidence post transplantation—mTOR-I + CNI vs. CNI

**Fig 3:**
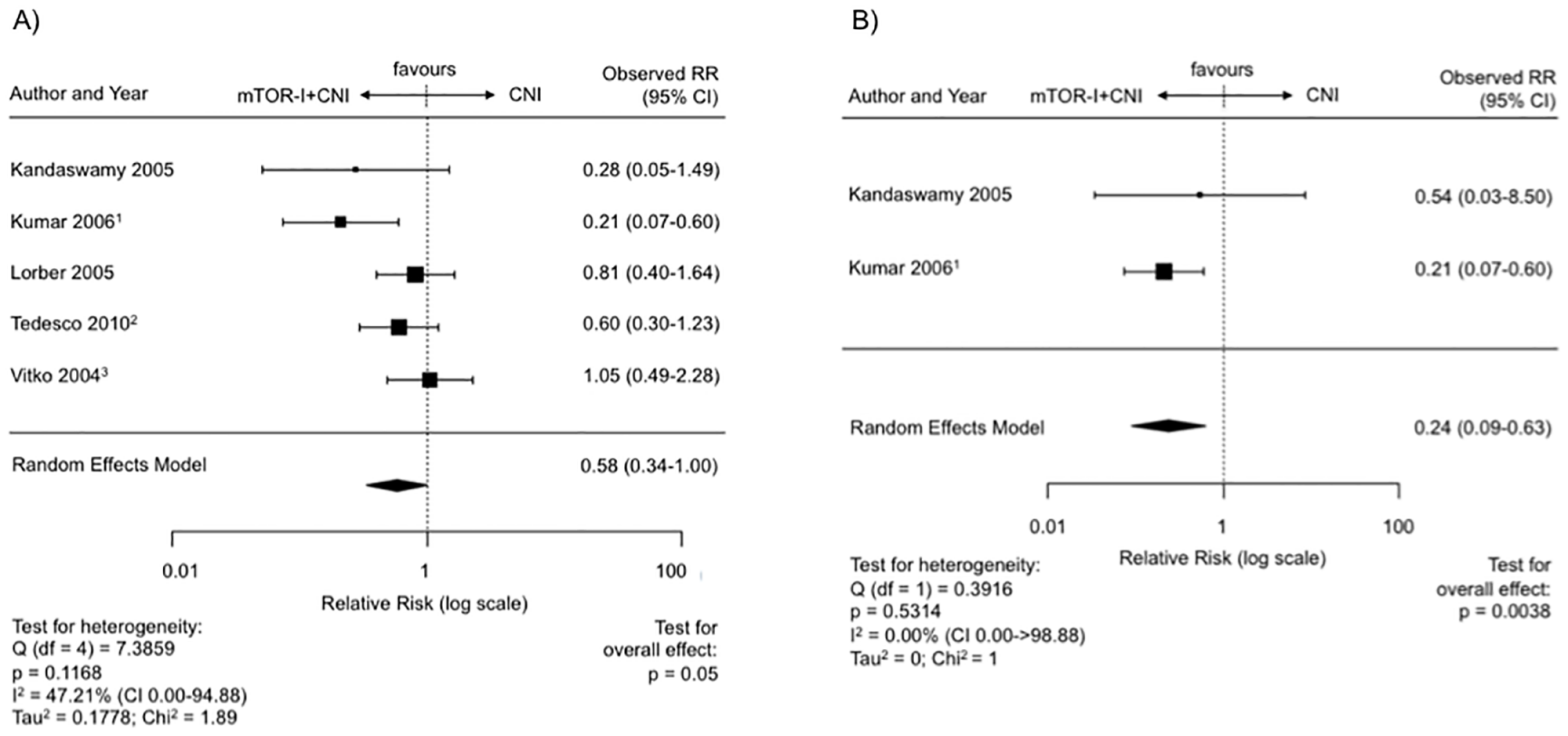
Malignancies on mTOR-I+CNI vs. CNI treatment post transplantation. (A) Forest plot indicating the relative risk of the occurence of malignancies. ^1^ Last update by Kumar et al. in 2008. ^2^ Last update by Cibrik et al. 2013. ^3^ Last update by Vitko in 2005. (B) Forest plot indicating the relative risk of the occurence of malignancies excluding NMSC’s. ^1^ Latest update by Kumar et al. in 2008.

RCTs with a combination therapy (mTOR-I+CNI, n = 5, SIR = 2, ERL = 3) showed a significantly reduced risk for the tumor incidence in comparison to CNI therapy (RR 0.58, CNI 0.34–1.00, p = 0.05) (Fig 3A). There was also no funnel plot asymmetry (p = 0.48) (S3 Fig) or significant heterogeneity observed between RCTs (I^2^ = 47.21%, Q-Test for heterogeneity: p = 0.12).

After exclusion of NMSCs (n = 2) there was also a significant difference (RR 0.24, CI 0.09–0.63, p = 0.0038) (Fig 3B). There was no indication of publication bias in the funnel plot as indicated by the regression test (p = 1.0) (S3 Fig). Also heterogeneity between the studies was not significant (I^2^ = 0.00%, Q-Test for heterogeneity: p = 0.53).

### Tumor incidence post transplantation—mTOR-I vs. CNI (monotherapy or combined with CNI)

**Fig 4:**
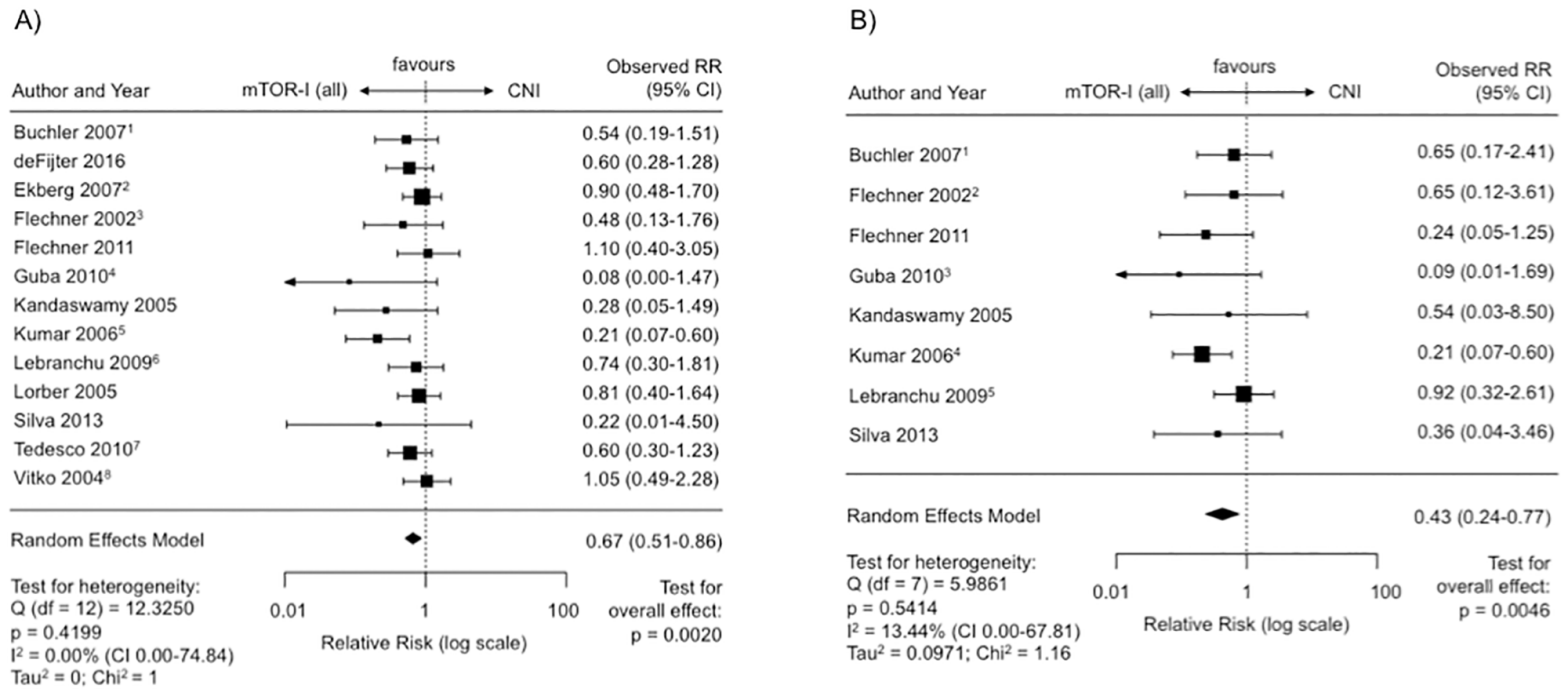
Malignancies on mTOR-I (monotherapy or combined with CNIs) versus CNI treatment post transplantation. (A) Forest plot indicating the relative risk of the occurence of malignancies. ^1^ Latest update by Fischer et al. in 2015. ^2^ Latest update by Ekberg et al. in 2009. ^3^ Latest update by Flechner et al. in 2007. ^4^ Latest update by Guba et al. in 2012. ^5^ Latest update by Kumar et al. in 2008. ^6^ Latest update by Lebranchu et al. in 2011. ^7^ Last update by Cibrik et al. 2013. ^8^ Latest update by Vitko et al. in 2005. (B) Forest plot indicating the relative risk of the occurence of malignancies excluding NMSC’s. ^1^ Last update by Gatault et al. in 2016. ^2^ Last update by Flechner et al. in 2007. ^3^ Last update by Guba et al. in 2012. ^4^ Last update by Kumar et al. in 2008. ^5^ Last update by Lebranchu et al. in 2011.

Taken together all studies on longterm tumor incidence (n = 13, SIR = 9, ERL = 4), the risk of posttransplant malignancy was significantly reduced under mTOR-I treatment (RR 0.67, CNI 0.51–0.86, p = 0.002) (Fig 4A). Heterogeneity between the studies was not significant (I^2^ = 0.0%, Q-Test for heterogeneity: p = 0.42).

When NMSCs were excluded 8 RCTs could still be included in the statistical analysis. Here, the relative risk was also significantly reduced under mTOR-Is (RR 0.43, CI 0.24–0.77, p = 0.0046) (Fig 4B). There was no funnel plot asymmetry (p = 0.55) (S3 Fig) or significant heterogeneity between RCTs (I^2^ = 13.44%, Q-Test for heterogeneity: p = 0.54).

### Graft survival (censored for death)—mTOR-I vs. CNI

There were 7 RCTs included in this analysis. SIR was the mTOR-I used in 6 RCTs and EVRL in 1 RCT.

**Fig 5:**
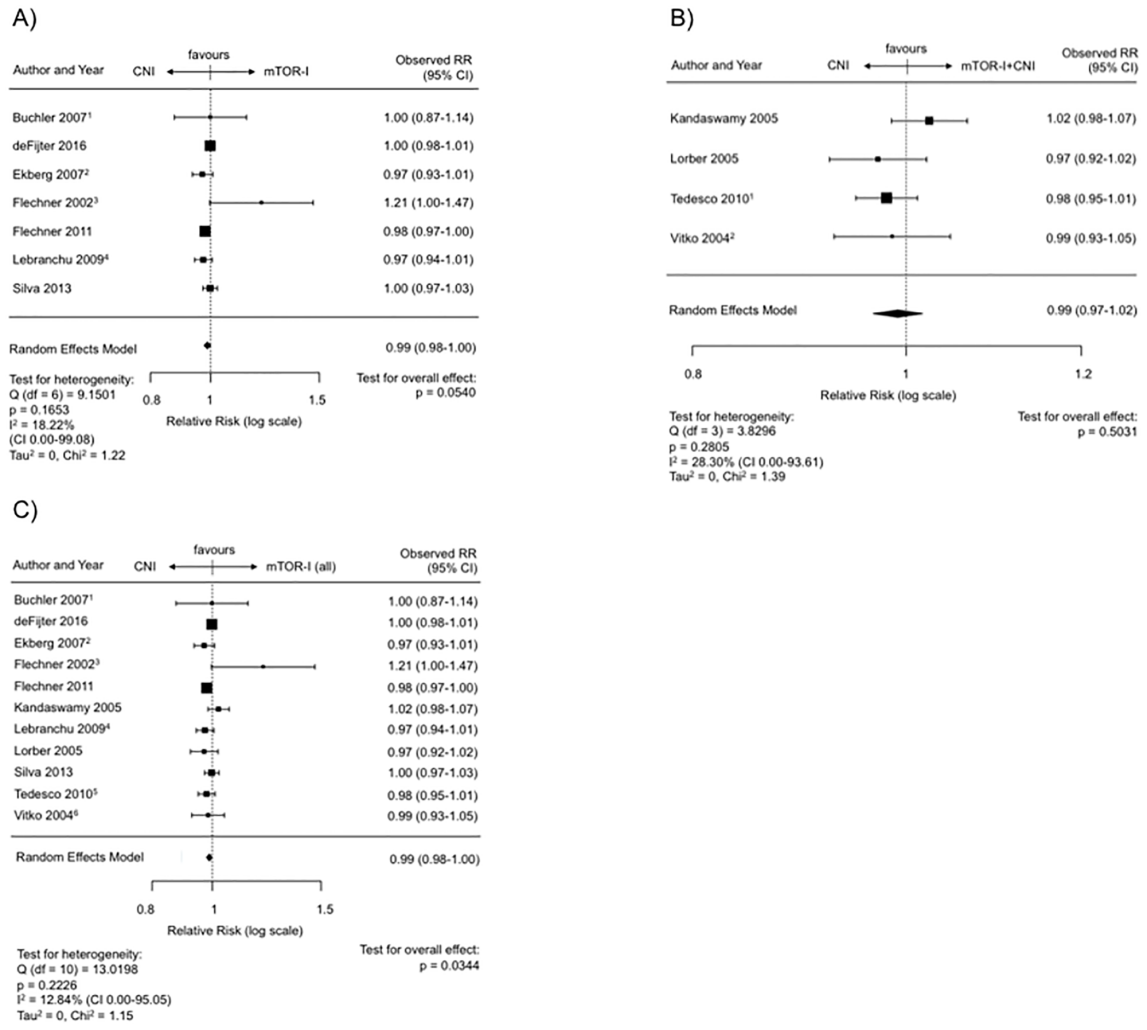
Graft survival censored for death post transplantation. (A) Forest plot indicating the graft survival censored for death on mTOR-I vs. CNI treatment. ^1^ Latest update by Gatault et al. in 2016. ^2^ Latest update by Ekberg et al. in 2009. ^3^ Latest update by Flechner et al. in 2007. ^4^ Latest update by Lebranchu et al. in 2011. (B) Forest plot indicating the graft survival censored for death on mTOR-I+CNI vs. CNI. ^1^ Last update by Cibrik et al. 2013. ^2^ Latest update by Vitko et al. in 2005. (C) Forest plot indicating the graft survival censored for death on mTOR-I (all) vs. CNI. ^1^ Latest update by Gatault et al. in 2016. ^2^ Latest update by Ekberg et al. in 2009. ^3^ Latest update by Flechner et al. in 2007. ^4^ Latest update by Lebranchu et al. in 2011. ^5^ Last update by Cibrik et al. 2013. ^6^ Latest update by Vitko et al. in 2005.

The ensuing analysis implied a minimal negative effect for the mTOR-I therapy even though a statistical significance was closely missed (RR 0.99, CI 0.98–1.00, p = 0.054; Fig 5A). The regression test for funnel plot asymmetry was not significant (p = 0.56) (S4 Fig). There was no heterogeneity between the RCTs (I^2^ = 18.22%, Q-Test for heterogeneity: p = 0.17).

### Graft survival (censored for death)—mTOR-I + CNI vs. CNI

There were four RCTs (SIR = 1, EVRL = 3) which presented longterm graft survival data censored for patients’ death. The meta-analysis revealed an estimated combined RR of 0.99 (CI 0.97–1.02, p = 0.50; Fig 5B). There was no indication of publication bias in the funnel plot or the regression test for asymmetry (p = 0.75) (S4 Fig). Also there was no substantial heterogeneity between the studies (I^2^ = 28.30%, Q-Test for heterogeneity: p = 0.28).

### Graft survival (censored for death)—mTOR-I vs. CNI (monotherapy or combined with CNI)

Taken together all studies with an mTOR-I-treatment arm (monotherapy or in combination with a CNI) compared to a CNI-based treatment (n = 11, SIR = 7, ERL = 4), the overall graft survival was statistically superior under CNIs (RR = 0.99; CI 0.98–1.00, p = 0.034) (Fig 5C). Similar to the analysis of mTOR-I without CNIs vs. CNIs (Fig 5A) the difference was minute and thus may not have been clinically relevant. There was no indication of publication bias in the funnel plot as indicated by the regression test (p = 0.28) (S4 Fig) and no heterogeneity between studies (I^2^ = 12.84%, Q-Test for heterogeneity: p = 0.22).

### Patient survival post transplantation—mTOR-I vs. CNI

There were 8 RCTs included in this analysis, which compared an mTOR-I- with a CNI-based treatment. Here, the mTOR-I was given without a CNI. SIR was the mTOR-I used in 7 and EVRL in 1 RCTs.

**Fig 6:**
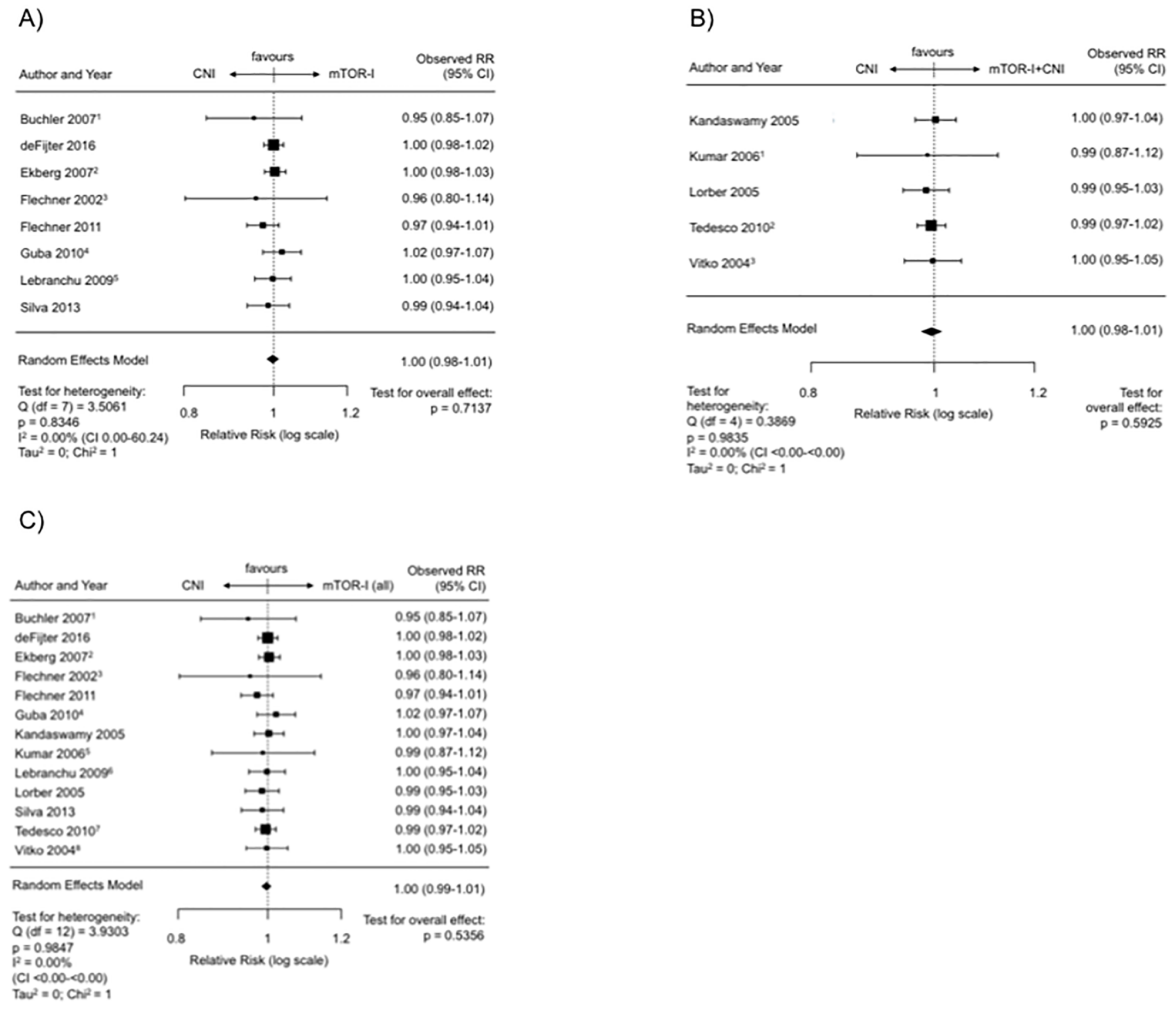
Overall patient survival post transplantation. (A) Forest plot indicating the overall patient survival on mTOR-I vs. CNI treatment. ^1^ Latest update by Gatault et al. in 2016. ^2^ Latest update by Ekberg et al. in 2009. ^3^ Latest update by Flechner et al. in 2007. ^4^ Latest update by Guba et al. in 2012. ^5^ Latest update by Lebranchu et al. in 2011. (B) Forest plot indicating the overall patient survival on mTOR-I+CNI vs. CNI treatment. ^1^ Latest update by Kumar et al. in 2008. ^2^ Last update by Cibrik et al. 2013. ^3^ Latest update by Vitko et al. in 2005. (C) Forest plot indicating the overall patient survival on mTOR-I (all) vs. CNI treatment. ^1^ Latest update by Gatault et al. in 2016. ^2^ Latest update by Ekberg et al. in 2009. ^3^ Latest update by Flechner et al. in 2007. ^4^ Latest update by Guba et al. in 2012. ^5^ Latest update by Kumar et al. in 2008. ^6^ Latest update by Lebranchu et al. in 2011. ^7^ Last update by Cibrik et al. 2013. ^8^ Latest update by Vitko et al. in 2005.

The mTOR-I showed a combined estimated RR of 1.00 (CI 0.98–1.01, p = 0.71) compared with the CNI treatment (Fig 6A). The regression test for funnel plot asymmetry was not significant (p = 0.40) (S4 Fig). There was no indication of a significant heterogeneity between the studies (I^2^ = 0.00%, Q-Test for heterogeneity: p = 0.83).

### Patient survival post transplantation—mTOR-I + CNI vs. CNI

Five RCTs (SIR = 2, EVRL = 3) presented longterm patient survival data in this setting. The meta-analysis revealed an estimated combined RR of 1.00 (CI 0.98–1.01, p = 0.60; Fig 6B). There was no indication of publication bias in the funnel plot or the regression test for asymmetry (p = 0.82) (S4 Fig). Also there was no substantial heterogeneity between the studies (I^2^ = 0.00%, Q-Test for heterogeneity: p = 0.98).

### Patient survival post transplantation—mTOR-I vs. CNI (monotherapy or combined with CNI)

When all studies with an mTOR-I-treatment arm either in monotherapy or in combination with a CNI were taken together and compared to a CNI-based treatment (n = 13, SIR = 9, ERL = 4), the overall patient survival showed a RR of 1.00 (CI 0.99–1.01, p = 0.54; Fig 6C). There was no indication of publication bias in the funnel plot as indicated by the regression test (p = 0.25) (S4 Fig). There was also no heterogeneity (I^2^ = 0.00%, Q-Test for heterogeneity: p = 0.98).

## Discussion

In this analysis the clinical evidence for a reduced incidence of malignancies under mTOR-Is compared to CNIs was summarized performing a systematic review and meta-analyses on the most recent and relevant high-quality randomized controlled trials in renal transplantation. As additional determinants graft and patient survival were analyzed. The results for the patient survival were of particular interest because a few papers had indicated a poorer survival under mTOR-I therapy in the recent years [20–23].

Importantly, only data from trials with a minimum follow-up of 24 months, averaging 41 months were included trying to elaborate the effect on malignancy and survival. Thus n = 5924 randomized pts. could be included making this analysis the largest of its kind with the longest follow-up to this topic.

### Malignancy

The overall tumor incidence in the immunosuppressed transplant patients is 2–4 times higher compared to the healthy general population [9, 10, 31–33].

We found a significantly reduced incidence of malignancy for an mTOR-I-based immunosuppression. This effect remained stable even in combination with CNIs and when all trials with an mTOR-I-based treatment (with or without CNI) were analyzed together.

This was expected and in part shown by others before [23, 34] since mTOR-Is have a particularly strong and well established effect against NMSCs [35, 36]. Squamous cell and basal cell carcinoma are the most frequent NMSCs (>90% of all skin cancers under immunosuppression). These tumors affect ultimately 50% or more of the white transplant recipients and are thus among the most common tumors under immunosuppression [37]. CsA has been associated with an increased NMSC incidence [38, 39].

Thus the more intriguing part was the next set of analyses for which NMSCs were excluded. For this, six studies could be included comparing mTOR-I without CNI vs. CNI. Another two trials with a comparison mTOR-I + CNI vs. CNI could be added.

Taken all these analyses together the RR for malignancy w/o NSMCs was substantially and significantly reduced (8 trials; RR 0.43, CI 0.24–0.77, p = 0.0046).

Over the past decade a series of meta-analyses and large population-based trials tried to elucidate the mTOR-I anti-tumor effect and outline a certain pattern for cancer incidences after solid organ transplantation [7–9, 40].

One of the first, Kauffman et al. published registry data from the OPTN/UNOS on this topic in 2005 [36]. At that time n = 2825 pts. treated with mTOR-Is either with or without CNIs had been reported to the registry. Their conclusion was a significant reduction of NMSCs and non-skin tumors. One problem here was that SRL users were defined only by the immunosuppressive regimen at discharge from the hospital and a tumor incidence of 0% in > 500 pts. under mTOR-Is over a follow up of close to 3 years may indicate underreporting.

In the first meta-analysis on mTOR-I vs. CNIs, no significant difference with respect to tumor incidence could be reported [41]. Limiting factor here was that only four RCTs with n = 447 could be included.

More recently, a meta-analysis combined with registry-data showed a 51% reduction of NMSCs under a SRL-based compared to a CNI-based immunosuppression for renal transplant recipients while failing to show a benefit with respect to other tumors [42]. Here, included RCTs reached back as far as 1999 including mTOR-I regimen no longer used nowadays. Follow-up of the included trials ranged from 6 months to 5 years averaging only 2 years. This may have been simply too short to detect significant effects on tumor incidence.

Similar data were reported by another meta-analysis on patients after renal transplantation receiving SRL [23]. Here, NMSCs were significantly reduced by 56% under SRL. The effect against “tumors other than NMSCs” was ambiguous: when all trials were analyzed together no effect was seen. Only in subgroup analyses, i.e. on “conversion trials” was a significantly reduced tumor incidence detectable. These divergent data indicate that certain mTOR-I regimen may be more beneficial against tumors than others. Inclusion of “early” trials dating back to 1999, when higher mTOR-I concentrations and loading doses were common, may have been responsible for the differences to our analysis.

Contrary to the above mentioned papers we tried to be more stringent about the study compilation including only trials with currently used mTOR-I regimen and comparable concentrations.

A Collaborative Transplant Study (CTS)- analysis on n = 78146 first deceased-donor kidney transplant recipients with a mean follow-up of 4.2 yrs provided more detailed information on NMSCs [34]. Similar to the other registry analyses the overall incidence of NMSCs but not “other” tumors was significantly reduced. For this trial, only de novo mTOR-I use was included and no difference was made between the patients receiving a CNI-free mTOR-I based regimen or the combination of the two.

The problems inherent to registry data are well known. And they may even be aggravated for informations which are not the primary focus of the registry. This was recently confirmed by a comparison of the SRTR with 15 linked cancer registries. Even though US transplant centers are required to report cancers in transplant recipients to the transplant network, this analysis revealed an extraordinary lack of accuracy and completeness for the SRTR [43]. The authors reported an estimated sensitivity for identifying cancer of only 52.5%.

For which tumor entities, other than NMSCs mTOR-Is have a beneficial effect we cannot tell by this analysis. Furthermore, we cannot exclude the possibility that mTOR-Is are permissive for some tumor entities while inhibiting others as has been postulated for prostate and renal cancers [42].

### Graft survival

Our analyses found a statistically significant benefit for CNIs vs. mTOR-Is w/o CNIs. The RR was 0.99 and thus a clinical relevance questionable. For the combination of mTOR-Is and CNIs no such effect was seen. It has been repetitively shown that a *de novo* or an early “monotherapy” with an mTOR-I results in a higher percentage of BPARs and a high number of therapy dropouts [44–47]. These problems may be avoided by using the combination of two [48–50] as is confirmed by our analyses.

### Patient survival

Another important aspect of our analysis is that there is no difference in patient survival (mTOR-I vs. CNI therapy). This is contrary to some reports of the recent years which indicated a potential threat for patients under mTOR-Is [20–23, 51]. The two most recent reports contained registry data from ANZDATA [20] and SRTR [51] including patients reaching as far back as 1996 and 2000 respectively. Despite the large number of patients and sophisticated statistical calculation models which can compensate for certain shortcomings, registry data may just not be appropriate to correctly analyze the survival in this context. Many patients are put on mTOR-Is for a history of or current malignancy and transplant dysfunction due to CAN—both situations for which an earlier death would be expected. Furthermore, many patients had been included in earlier years when higher doses of mTOR-Is were standard. Without doubt “longterm” data from randomized controlled trials are the most accurate to receive a correct answer on the question of survival. The only trial to date that used randomized controlled data and showed a worse survival under mTOR-Is was the meta-analysis by Knoll et al. [23]. Here, a 43% increased risk of death under SRL for renal transplant patients was reported. Trial composition may have been at least in part responsible for these data. Most of the included trials used SRL *de novo* (76%) and often with extraordinary high loading and maintenance doses. Importantly, mortality under “low dose” SRL, as is preferably used nowadays in transplantation was not increased.

### Limitations

First, we did not have patient level information from the included RCTs. Second, malignancy and survival were the primary endpoint in some but not all of the RCTs. However, survival as the most important outcome variable has certainly been documented accurately. This most likely holds true also for malignancy events. Post-transplant malignancy and the as of yet unclear significance of an mTOR-I therapy is a highly relevant aspect. Third, the trials included patients with varying risk of malignancy: some excluded those with any cancer, while others excluded only those with a history of non-skin cancer.

### Conclusions

Early initiation or conversion to mTORI-I within 3 months of kidney transplantation may reduce the future risk of cancer, when compared with patients remaining on CNI-based regimens. The primary effect is against NMSCs but there also exists a significant effect against other tumors. The predominant part of the anti-tumor effect remains present even when administered in combination with a CNI. There is no increased mortality nor graft loss under currently used mTOR-I based regimen.

## Supporting information

S1 Table Included trials mTOR-I vs. CNI. (DOCX)

S2 Table Included trials mTOR-I + CNI vs. CNI. (DOCX)

S3 Table PRISMA checklist. (DOC)

S1 Fig Cochrane Collaboration’s tool for trials mTOR-I vs. CNI. (TIF)

S2 Fig Cochrane Collaboration’s tool for trials mTOR-I + CNI vs. CNI. (TIF)

S3 Fig Funnel plots malignancy. (TIF)

S4 Fig Funnel plots graft and patient survival. (TIF)

## Abbreviations

AC: allocation concealment
CAN: chronic allograft nephropathy
CNI: Calcineurininhibitor
CsA: Cyclosporine
CTS: Collaborative Transplant Study
EVRL: Everolimus
ITT: Intention to treat
mTOR-I: mTOR-Inhibitor
NMSC: non-melanoma skin cancer
OPTN: Organ Procurement and Transplantation Network
SRL: Sirolimus
SRTR: Scientific Registry of Transplant Recipients
TAC: Tacrolimus
