# Supplementary material for: Effects of mTOR-Is on malignancy and survival following renal transplantation: A systematic review and meta-analysis of randomized trials with a minimum follow-up of 24 months
Source: PLoS One. 2018 Apr 16;13(4):e0194975. doi: 10.1371/journal.pone.0194975 (PMC5901925; doi:10.1371/journal.pone.0194975)
Supplement: S1 Table — (DOCX) [file pone.0194975.s001.docx]

| **mTOR-I vs. CNI**  **Trial** | **Therapy** | **Induction** | **mTOR-I (pts.)** | **all CNI (pts.)** | **mTOR-I initiation** | **Duration of study (month)** | **Follow-Up longterm (month)** | **Patient survival (%)** | **Graft survival**  **– censored for death (%)** | **Tumor incidence**  **- all**  **longterm (%)** | **Tumor incidence**  **- no NMSC**  **longterm (%)** | **Jadad**  **(0-5)** | **ITT** |
| --- | --- | --- | --- | --- | --- | --- | --- | --- | --- | --- | --- | --- | --- |
| Buchler 2007,  Lebranchu 2012,  Gatault 2016 (1-3)  (RTx) | Sir CsA | ATG ATG | 71 | 74 | de novo | 12 | 60 (96) | 97 (12mo), 93 (60mo), 86,3 (96mo) 97 (12mo), 93 (60mo), 90,6 (96mo) | 93 (12mo), 87 (60mo), 85,5 (96mo) 96 (12mo), 91 (60mo), 85,5 (96mo) | 7  13 (no difference at 96 month ) | 4,8  7,4 (no difference at 96 month ) | 3 | YES |
| deFijter (4)  2016  (RTx) | Ev+MPA CNI+MPA | Basiliximab Basiliximab | 359 | 356 | 10-14 weeks post Tx | 24 | 24 | 98,0 (12mo), 97,4 (24mo) 98,5 (12mo), 97,4 (24mo) | 99,4 (12mo), 98,8 (24mo) 98,8 (12mo), 98,9 (24mo) | 2,8  4,7 | NR | 3 | YES |
| Ekberg 2007,  Ekberg 2009 (5, 6)  (RTx) | Standard CsA  Low CsA  Low Tac  Low Sir | None Daclizumab Daclizumab Daclizumab | 399 | 1190 | de novo | 12 | 36 | 96,5 (12mo), 96 (24mo), 94 (36mo) 98,2 (12mo), 98 (24mo), 95 (36mo) 97,2 (12mo), 96 (24mo), 95 (36mo) 96,8 (12mo), 95 (24mo), 95 (36mo) | 91,9 (12mo), 91 (24mo), 91 (36mo) 94,3 (12mo), 91 (24mo), 91 (36mo) 96,4 (12mo), 95 (24mo), 93 (36mo) 91,7 (12mo), 91 (24mo), 89 (36mo) | 4 3 3 3  (month 12-36) | NR | 3 | YES |
| Flechner 2002,  Flechner 2007 (7, 8)  (RTx) | Sir  CsA | Basiliximab Basiliximab | 31 | 30 | de novo | 12 | 60 | 96,7 (12mo), 87,1 (60mo) 100 (12mo), 91 (60mo) | 96,4 (60mo) 79,7 (60mo) | 9,7 20 | 6,5 10 | 3 | YES |
| Flechner (9)  2011  (RTx) | Sir  Sir+MPA Tac+MPA | Daclizumab Daclizumab Daclizumab | 152 152 | 139 | de novo | 12 | 24 | 97,3 (12mo), 94,4 (24mo) 95,2(12mo), 94,5 (24mo) 97,0 (12mo), 97,0 (24mo) | n.r. (12mo), 98,7 (24mo) n.r. (12mo), 97,4 (24mo) 100 (12mo), 100 (24mo) | 4,6 3,3 3,6 | 0,7 0,7 2,9 | 3 | YES |
| Guba 2010,  Guba 2012 (10, 11)  (RTx) | Sir  CsA | ATG-F ATG-F | 69 | 71 | 14-21 d post Tx | 12 | 36 | 99 (12mo), 99 (36mo) 99 (12mo), 97 (36mo) | NR | 0 8 | 0 7 | 3 | YES |
| Lebranchu 2009, Servais 2009,  Lebranchu 2011 (12-14)  (RTx) | Sir CsA | Daclizumab Daclizumab | 95 | 97 | 3 mo  post Tx | 12 | 48 | 99,5 (12mo), 97,4 (48mo) 97,9 (12mo), 97,6 (48mo) | 97,4 (48mo) 100 (48mo) | 7,8 10,6  (month 12-48) | 6,5 7,1  (month 12-48) | 3 | YES |
| Silva (15)  2013  (RTx) | Sir+MPA Tac+MPA | Basiliximab Basiliximab | 97 | 107 | 3 mo  post Tx | 24 | 24 | 95,9 (24mo) 97,2 (24mo) | 99,0 (24mo) 99,1 (24mo) | 0 1,9 | 0 1,9 | 3 | YES |

1. Buchler M, Caillard S, Barbier S*, et al*: Sirolimus versus cyclosporine in kidney recipients receiving thymoglobulin, mycophenolate mofetil and a 6-month course of steroids. Am J Transplant 7: 2522-2531, 2007.

2. Lebranchu Y, Snanoudj R, Toupance O*, et al*: Five-year results of a randomized trial comparing de novo sirolimus and cyclosporine in renal transplantation: the SPIESSER study. Am J Transplant 12: 1801-1810, 2012.

3. Gatault P, Bertrand D, Buchler M*, et al*: Eight-year results of the Spiesser study, a randomized trial comparing de novo sirolimus and cyclosporine in renal transplantation. Transpl Int 29: 41-50, 2016.

4. de Fijter JW, Holdaas H, Oyen O*, et al*: Early Conversion From Calcineurin Inhibitor- to Everolimus-Based Therapy Following Kidney Transplantation: Results of the Randomized ELEVATE Trial. Am J Transplant 2016.

5. Ekberg H, Tedesco-Silva H, Demirbas A*, et al*: Reduced exposure to calcineurin inhibitors in renal transplantation. N Engl J Med 357: 2562-2575, 2007.

6. Ekberg H, Bernasconi C, Tedesco-Silva H*, et al*: Calcineurin inhibitor minimization in the Symphony study: observational results 3 years after transplantation. Am J Transplant 9: 1876-1885, 2009.

7. Flechner SM, Goldfarb D, Solez K*, et al*: Kidney transplantation with sirolimus and mycophenolate mofetil-based immunosuppression: 5-year results of a randomized prospective trial compared to calcineurin inhibitor drugs. Transplantation 83: 883-892, 2007.

8. Flechner SM, Goldfarb D, Modlin C*, et al*: Kidney transplantation without calcineurin inhibitor drugs: a prospective, randomized trial of sirolimus versus cyclosporine. Transplantation 74: 1070-1076, 2002.

9. Flechner SM, Glyda M, Cockfield S*, et al*: The ORION study: comparison of two sirolimus-based regimens versus tacrolimus and mycophenolate mofetil in renal allograft recipients. Am J Transplant 11: 1633-1644, 2011.

10. Guba M, Pratschke J, Hugo C*, et al*: Renal function, efficacy, and safety of sirolimus and mycophenolate mofetil after short-term calcineurin inhibitor-based quadruple therapy in de novo renal transplant patients: one-year analysis of a randomized multicenter trial. Transplantation 90: 175-183, 2010.

11. Guba M, Pratschke J, Hugo C*, et al*: Early conversion to a sirolimus-based, calcineurin-inhibitor-free immunosuppression in the SMART trial: observational results at 24 and 36months after transplantation. Transpl Int 25: 416-423, 2012.

12. Lebranchu Y, Thierry A, Toupance O*, et al*: Efficacy on renal function of early conversion from cyclosporine to sirolimus 3 months after renal transplantation: concept study. Am J Transplant 9: 1115-1123, 2009.

13. Lebranchu Y, Thierry A, Thervet E*, et al*: Efficacy and safety of early cyclosporine conversion to sirolimus with continued MMF-four-year results of the Postconcept study. Am J Transplant 11: 1665-1675, 2011.

14. Servais A, Meas-Yedid V, Toupance O*, et al*: Interstitial fibrosis quantification in renal transplant recipients randomized to continue cyclosporine or convert to sirolimus. Am J Transplant 9: 2552-2560, 2009.

15. Silva HT, Jr., Felipe CR, Garcia VD*, et al*: Planned randomized conversion from tacrolimus to sirolimus-based immunosuppressive regimen in de novo kidney transplant recipients. Am J Transplant 13: 3155-3163, 2013.
