# Supplementary material for: Effects of mTOR-Is on malignancy and survival following renal transplantation: A systematic review and meta-analysis of randomized trials with a minimum follow-up of 24 months
Source: PLoS One. 2018 Apr 16;13(4):e0194975. doi: 10.1371/journal.pone.0194975 (PMC5901925; doi:10.1371/journal.pone.0194975)
Supplement: S2 Table — (DOCX) [file pone.0194975.s002.docx]

| **mTOR-I + CNI vs. CNI**  **Trial** | **Therapy** | **Induction** | **all CNI (pts.)** | **mTOR-I + CNI (pts.)** | **mTOR-I initiation** | **Duration of study (month)** | **Follow-Up longterm (month)** | **Patient survival (%)** | **Graft survival**  **– censored for death (%)** | **Tumor incidence**  **- all**  **longterm (%)** | **Tumor incidence**  **- no NMSC**  **longterm (%)** | **Jadad**  **(0-5)** | **ITT** |
| --- | --- | --- | --- | --- | --- | --- | --- | --- | --- | --- | --- | --- | --- |
| Kandaswamy 2005 (1)  (RTx) | CsA+MPA hi Tac+Sir 2,0 lo Tac+Sir 5,0 | Thymoglobulin Thymoglobulin Thymoglobulin | 85 | 72 82 | de novo | 24 | 24 | 98 (6mo), 98 (12mo), 98 (24mo) 97 (6mo), 97 (12mo). 97 (24mo) 99 (6mo), 97 (12mo), 97 (24mo) | 99 (6mo), 99 (12mo), 97 (24mo) 97 (6mo), 97 (12mo), 97 (24mo) 96 (6mo), 99 (12mo), 99 (24mo) | 4,7 0 2,4 | 1,2 0 1,2 | 2 | YES |
| Kumar 2006,  Kumar 2008  (2, 3)  (RTx) | Sir+CsA Sir+Tac  CsA+MPA  Tac+MPA | Basiliximab Basiliximab Basiliximab Basiliximab | 50 50 | 50 50 | de novo / 2d post Tx | 60 | 96 | 82 (60mo)  82 (60mo) 82 (60mo) 84 (60mo) | NR | 4 4 18 20 | 4 4 18 20 | 2 | NO |
| Lorber 2005 (4)  (RTx) | Ev1,5+CsA red Ev3,0+CsA red CsA st+MPA | None | 196 | 193 194 | de novo | 12 | 36 | 96,9 (12mo), 93,8 (36mo) 96,4 (12mo), 93,3 (36mo) 98 (12mo), 94,9 (36mo) | 91,2 (12mo), 88,1 (36mo) 95,9 (12mo), 92,3 (36mo) 94,9 (12mo), 92,9 (36mo) | 4,7 5,2 6,1 | NR | 3 | YES |
| Tedesco 2010,  Cibrik 2013 (5, 6)  (RTx) | Ev1,5+CsA red Ev3,0+CsA red CsA st+MPA | Basiliximab Basiliximab Basiliximab | 277 | 277 279 | de novo | 24 | 24 | 97,5 (12mo), 96,8 (24mo) 96,8 (12mo), 96,4 (24mo) 97,8 (12mo), 97,1 (24mo) | 95,7 (12mo), 94,2 (24mo) 95,3 (12mo), 95,9 (24mo) 96,8 (12mo), 96,0 (24mo) | 8,0 / 3,3 5,4 / 2,5 8,8 / 4,8  (neoplasm / malignancy) | NR | 3 | YES |
| Vitko 2004,  Vitko 2005  (7, 8)  (RTx) | Ev1,5+CsA red Ev3,0+CsA red CsA st+MPA | None | 196 | 194 198 | de novo | 12 | 36 | 95,4 (6mo), 94,8 (12mo), 92,3 (36mo) 96,5 (6mo), 96 (12mo), 90,9 (36mo) 98,5 (6mo), 97,4 (12mo), 91,8 (36mo) | 96,4 (6mo), 95,4 (12mo), 92,8 (36mo) 91,4 (6mo), 89,4 (12mo), 83,3 (36mo) 92,3 (6mo), 90,8 (12mo), 89,3 (36mo) | 5,2 4,5 4,6 | NR | 3 | YES |

1. Kandaswamy R, Melancon JK, Dunn T*, et al*: A prospective randomized trial of steroid-free maintenance regimens in kidney transplant recipients--an interim analysis. Am J Transplant 5: 1529-1536, 2005.

2. Kumar MS, Heifets M, Moritz MJ*, et al*: Safety and efficacy of steroid withdrawal two days after kidney transplantation: analysis of results at three years. Transplantation 81: 832-839, 2006.

3. Anil Kumar MS, Irfan Saeed M, Ranganna K*, et al*: Comparison of four different immunosuppression protocols without long-term steroid therapy in kidney recipients monitored by surveillance biopsy: five-year outcomes. Transpl Immunol 20: 32-42, 2008.

4. Lorber MI, Mulgaonkar S, Butt KM*, et al*: Everolimus versus mycophenolate mofetil in the prevention of rejection in de novo renal transplant recipients: a 3-year randomized, multicenter, phase III study. Transplantation 80: 244-252, 2005.

5. Tedesco Silva H, Jr., Cibrik D, Johnston T*, et al*: Everolimus plus reduced-exposure CsA versus mycophenolic acid plus standard-exposure CsA in renal-transplant recipients. Am J Transplant 10: 1401-1413, 2010.

6. Cibrik D, Silva HT, Jr., Vathsala A*, et al*: Randomized trial of everolimus-facilitated calcineurin inhibitor minimization over 24 months in renal transplantation. Transplantation 95: 933-942, 2013.

7. Vitko S, Margreiter R, Weimar W*, et al*: Everolimus (Certican) 12-month safety and efficacy versus mycophenolate mofetil in de novo renal transplant recipients. Transplantation 78: 1532-1540, 2004.

8. Vitko S, Margreiter R, Weimar W*, et al*: Three-year efficacy and safety results from a study of everolimus versus mycophenolate mofetil in de novo renal transplant patients. Am J Transplant 5: 2521-2530, 2005.
