# Supplementary figures and images for: Effects of mTOR-Is on malignancy and survival following renal transplantation: A systematic review and meta-analysis of randomized trials with a minimum follow-up of 24 months
Source: PLoS One. 2018 Apr 16;13(4):e0194975. doi: 10.1371/journal.pone.0194975 (PMC5901925; doi:10.1371/journal.pone.0194975)

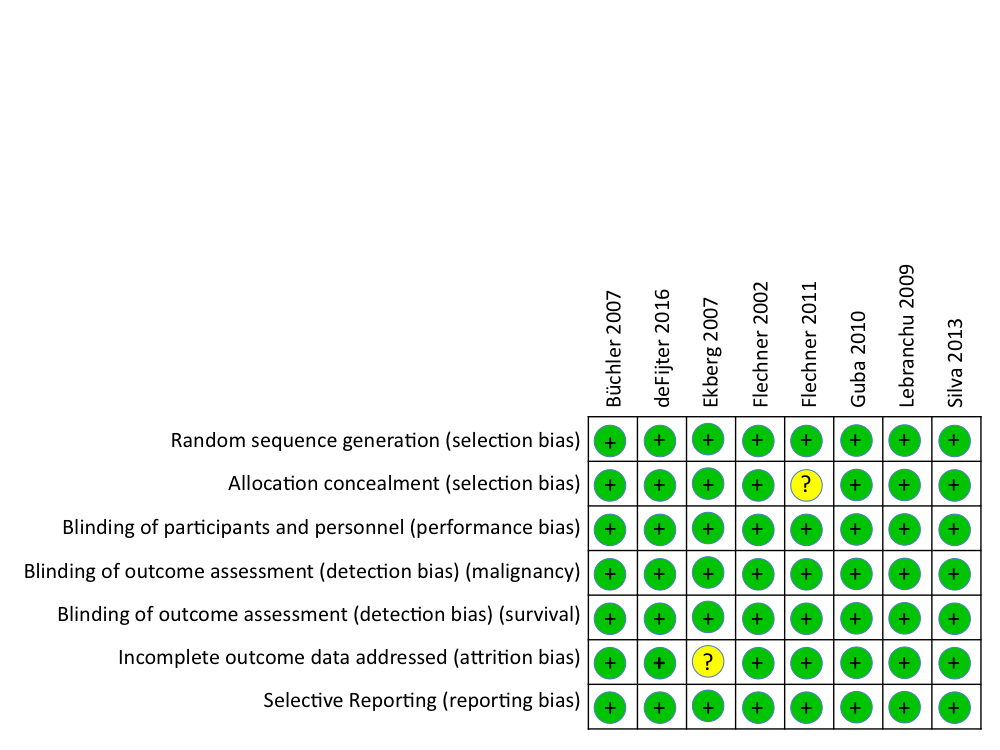

Supplement: S1 Fig — (TIF) [file pone.0194975.s004.tif]

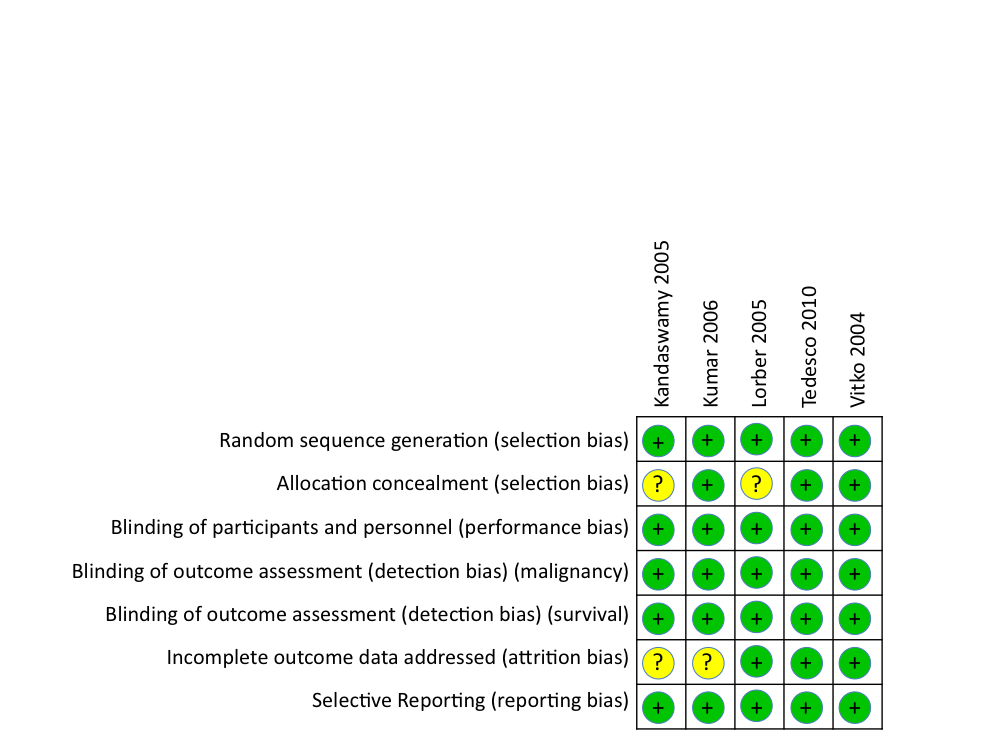

Supplement: S2 Fig — (TIF) [file pone.0194975.s005.tif]

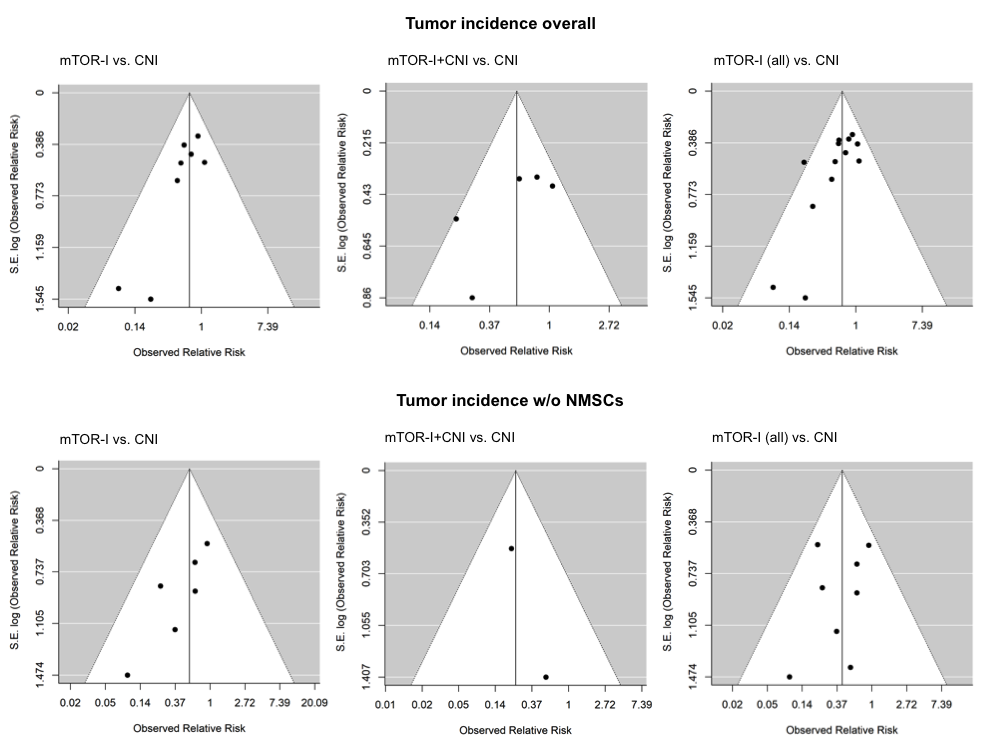

Supplement: S3 Fig — (TIF) [file pone.0194975.s006.tif]

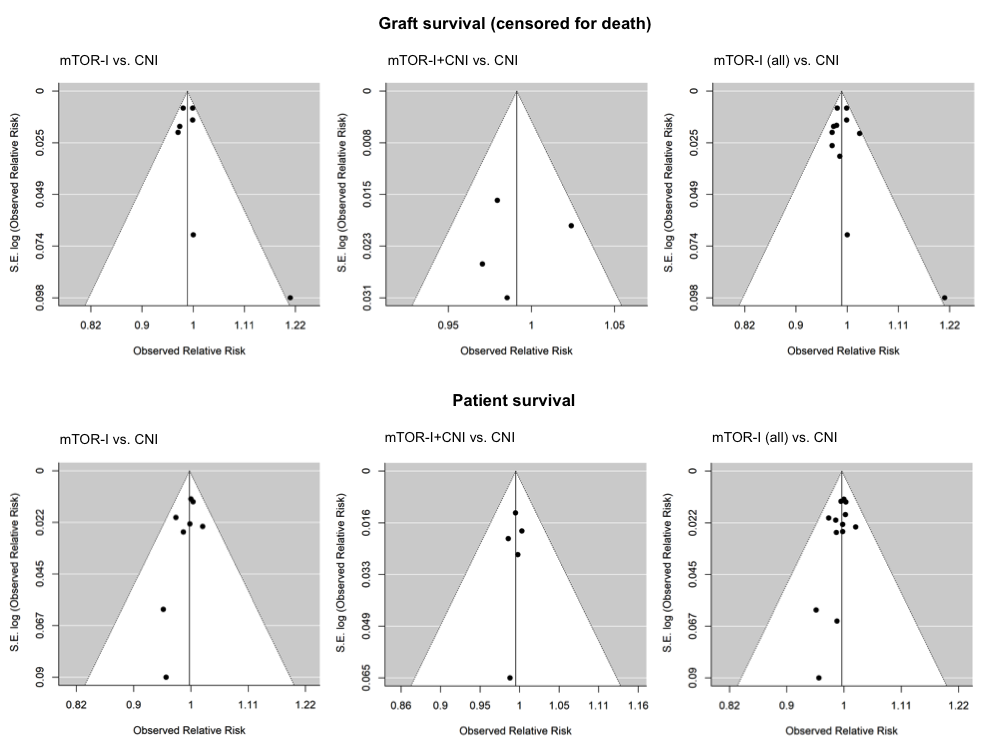

Supplement: S4 Fig — (TIF) [file pone.0194975.s007.tif]
